# Decreasing hospitalizations through geriatric hotlines: a prospective French multicenter study of people aged 75 and above

**DOI:** 10.1186/s12877-023-04495-9

**Published:** 2023-11-28

**Authors:** Luc Goethals, Nathalie Barth, Laure Martinez, Noémie Lacour, Magali Tardy, Jérôme Bohatier, Marc Bonnefoy, Cédric Annweiler, Caroline Dupre, Bienvenu Bongue, Thomas Celarier

**Affiliations:** 1 SAINBIOSE laboratory, U1059 INSERM - University of Jean Monnet, Saint-Etienne, France; 2 Chaire Santé des Ainés, University of Jean Monnet, Saint-Etienne, France; 3 Gerontopole Auvergne-Rhône-Alpes, Saint-Etienne, France; 4 Department of Clinical Gerontology, Saint-Etienne University Hospital, Saint-Etienne, France; 5 Department of Clinical Gerontology, Firminy Hospital, Firminy, France; 6 Department of Clinical Gerontology, Saint-Chamond Hospital, Saint-Chamond, France; 7 Department of Clinical Gerontology, Clermont-Ferrand University Hospital, Clermont-Ferrand, France; 8 Department of Clinical Gerontology, Lyon Sud University Hospital, Lyon, France; 9 Department of Geriatric Medicine and Memory Clinic, Research Center on Autonomy and Longevity, University Hospital of Angers, Angers, France; 10 UPRES EA 4638, University of Angers, Angers, France; 11 Department of Medical Biophysics, Schulich School of Medicine and Dentistry, Robarts Research Institute, University of Western Ontario, London, ON Canada; 12 Support and Education Technical Centre of Health Examination Centres (CETAF), Saint-Etienne, France

**Keywords:** Aged adults, Health care, Hotline, Emergency admission, General practitioner, Geriatrician

## Abstract

**Background:**

The Emergency unit of the hospital (Department) (ED) is the fastest and most common way for most French general practitioners (GPs) to respond to the complexity of managing older adults patients with multiple chronic diseases. In 2013, French regional health authorities proposed to set up telephone hotlines to promote interactions between GP clinics and hospitals. The main objective of our study was to analyze whether the hotlines and solutions proposed by the responding geriatrician reduced the number of hospital admissions, and more specifically the number of emergency room admissions.

**Methods:**

We conducted a multicenter observational study from April 2018 to April 2020 at seven French investigative sites. A questionnaire was completed by all hotline physicians after each call.

**Results:**

The study population consisted of 4,137 individuals who met the inclusion and exclusion criteria. Of the 4,137 phone calls received by the participants, 64.2% (*n* = 2 657) were requests for advice, and 35.8% (*n* = 1,480) were requests for emergency hospitalization. Of the 1,480 phone calls for emergency hospitalization, 285 calls resulted in hospital admission in the emergency room (19.3%), and 658 calls in the geriatric short stay (44.5%). Of the 2,657 calls for advice/consultation/delayed hospitalization, 9.7% were also duplicated by emergency hospital admission.

**Conclusion:**

This study revealed the value of hotlines in guiding the care of older adults. The results showed the potential effectiveness of hotlines in preventing unnecessary hospital admissions or in identifying cases requiring hospital admission in the emergency room. Hotlines can help improve the care pathway for older adults and pave the way for future progress.

**Trial registration:**

Registered under Clinical Trial Number NCT03959475. This study was approved and peer-reviewed by the Ethics Committee for the Protection of Persons of Sud Est V of Grenoble University Hospital Center (registered under 18-CETA-01 No.ID RCB 2018-A00609-46).

**Supplementary Information:**

The online version contains supplementary material available at 10.1186/s12877-023-04495-9.

## Introduction

 The aging of the population is a global phenomenon and a public health issue, particularly for healthcare systems [1, 2]. On January 1st, 2021, 9.5% of the French population was over 75 years of age, an increase of 2.3% compared to 2000 [3, 4]. According to the French National Institute of Statistics and Economic Studies (INSEE) [4], the proportion of people over 75 years old could exceed 16% in 2050. In 2050, France would have 4 million older adults with a loss of autonomy, i.e., 16.4% of the population aged 60 or more (compared to 15.3% in 2015). Highly dependent people would represent 4.3% of the population aged 60 or more (compared to 3.7% in 2015) [5].

The organizational model of hospitals is one of the first aspects affected by the aging of the population [6]. There is no doubt that older people will become the “core business” of the hospital. But how will the hospital cope with this problem? In France, the organization of geriatric services relies on the city’s main hospital to provide comprehensive care for older patients, notably through geriatric short-stay units, rehabilitation care units, healthcare outpatient teams, or even nursing homes [7]. In France, for general practitioners (GP), the Emergency Department (EDs) is the quickest and most common way to respond to the complexity of managing older adults patients with multiple pathologies [8, 9]. In addition to the observed overcrowding, studies have shown that the emergency department experience can have deleterious effects on the health of the older adults [10]. The EDs can be traumatic for the older adults and generate numerous complications, generally referred to as geriatric syndromes [11–15]. For emergency physicians, the management of older adults with multiple chronic pathologies, often unaccompanied by a relative, is a complex exercise, aggravated by the lack of time induced by increasingly overloaded emergency departments [6].

Studies have shown that 13–40% of case admissions to EDs were inappropriate and that ambulatory care or deferred hospitalization would have been possible with appropriate geriatric advice [16–19]. In addition, this large number of hospital admissions represents an economic challenge. According to the High Council for the Future of Health Insurance (HCAAM), the additional costs induced by the segmentation and incompatibility of proposed solutions, such as unjustified recourse to hospitalization, have been estimated at 2 billion euros [20]. These data highlight the need for better coordination between ambulatory medicine and hospitals for more appropriate access to care for older adults [21].

In order to improve the city/clinic-hospital interface, the Regional Health Agencies (RHA) initially organized the territory into a network centered on a hospital, integrating both emergency rooms and geriatric short-stay services allowing direct patient registration. The Geriatric Short Stay is composed of a medical and paramedical team, whose mission is to provide adapted care to a population of older patients with acute medical, psychological, and/or social problems. On this occasion, the RHA proposed the setting up of telephone hotlines to encourage GP clinic-hospital interactions. The main objective was to reduce the number of hospitalizations and improve the care pathways and health status of older patients. The physician answering the hotline belongs to the geriatric short-stay department. A telephone number is provided to the GPs and operates from Monday to Friday according to a schedule defined by the geriatric short-stay service. Professionals directly call a geriatric physician who can provide diagnostic, therapeutic, and orientation advice. This geriatric physician also helps to orient the patient in the geriatric pathway if necessary. The use of this telephone is heterogeneous across the territory. Its usefulness remains to be demonstrated, but it seems that hotlines are helpful for general practitioners, the main callers [22], and that they improve the care and management of older patients [23, 24].

The study’s main objective was to investigate the potential effectiveness of hotlines in preventing inappropriate emergency room visits by older adults through the solutions provided by the responding physicians (geriatricians).

## Materials and methods

### Study design and period

It was an observational, multicenter, and descriptive study conducted from April 2018 to April 2020 at seven French investigative sites (hospitals of St-Etienne, Clermont-Ferrand, Lyon-Sud, Bordeaux, Angers, Firminy, and Saint-Chamond).

Physicians receiving the hotline calls completed two questionnaires: the first when a call was received and the second when a patient arrived in the geriatric short-stay service after having been referred by the hotline. In this study, we focus on the first questionnaire (Appendix 1).

The selection criteria for study participants were to be aged 75 and older and participants living at home or in an institution (including independent living facilities). Patients under 75 were not included. These criteria were to be verified by the physician on hotline duties.

### Recorded variables and source data

For the first questionnaire, completed by geriatric physicians on the hotline, socio-demographic, administrative and health information were collected for the study. These data included the identification of both interlocutors, patient age, reasons of calling, perceived degree of emergency by the geriatric specialist (rated from 0 to 10; 0 being no urgency and 10 being absolute urgency), and the responses proposed by the geriatric specialist. The call duration (in minutes) was also recorded.

This questionnaire was anonymous with no identifying parameter. It was filled out at each call made via the hotline, regardless of the answers proposed by the physician (referral or not to short-stay geriatric care).

### Request and use of the hotline

Study participants use the hotline for different reasons. The first questionnaire identifies the main reason for the call. Reasons could have be: counseling, hospitalization in the emergency department (SAU), emergency hospitalization in geriatrics, a request for consultation, or deferred hospitalization.

For this study, we classified calls into two groups: emergency (general or geriatric emergency) and non-emergency (counseling, consultation, or deferred hospitalization). Upon reception of the call, the geriatrician on duty of the hotline will propose several solutions, from a simple advice to an hospitalization in a general emergency or geriatric service, through a deferred hospitalization, an outpatient mobile team consultation, a day hospitalization, a teleconsultation, or a temporary accommodation. If the prognosis was not life-threatening but needed hospitalization, they were offered a short-stay admission to a geriatric ward. However, sometimes there were no available beds in the short-stay geriatric, then the patient was sent to the emergency ward.

The geriatric physician answering the hotline call is the one deciding which is the best solution between admission to the emergency ward, hospitalization or outpatient care.

### Data management and analysis

IBM SPSS Statistics (version 29.0.1.0) was used for all statistical analyses. The study population included participants of the geriatric short stay unit answering calls. Calls received were classified and groups compared by chi-square or Wilcoxon rank-sum test (the emergency call group and the delayed advice, consultation, or hospitalization group). The solution offered by the hotline receiver could be concordant or discordant with the request of the GP calling. We created four groups of discordant and concordant calls/requests to analyze the characteristics of the individuals and the differences between the hotline centers. The concordant calls consisted of: request calls for advice that resulted by an advice given by the geriatrician; request calls for hospital admissions that received an offer in hospitalization in a geriatric emergency or in the emergency room by the geriatrician. The discordant calls were made up of calls whose response did not match the request made by the GP. Examples: request calls for advice that received an hospitalization as response; request call for hospitalization that was denied by the geriatrician. They allow describing the difference between the request of the call and the answer given. A *p* < 0.05 was considered statistically significant.

## Results

The study enrolled 4 137 participants fulfilling the inclusion and exclusion criteria. The caller was a general practitioner in 72.9% of times, and the respondent was a hospital practitioner in 70.7% of cases. The average age of the patients for whom the call was made was 86.8 years (SD = 5.6). The degree of emergency depicted by the responding physician was lower than that of the caller (5.44 (SD = 2.42 compared to 5.86 (SD = 2.34)). The final call duration was 8.9 min (SD = 7.45).

Of the 4 137 calls received, 64.2% (*n* = 2,657) were for advice and 35.8% (*n* = 1,480) were request for emergency hospital admissions (general or geriatric emergency department). The analysis of the two call groups showed differences (see Table 1). The degree of urgency was higher for emergency hospital admissions requests than that for advice, as perceived by both the caller and the responding physician (*p* < 0.0001). There was no effect of patient age on the main reason for the call (*p* = 0.470). Of the 1,480 calls for emergency hospital admissions, 285 calls resulted in hospital admission in the emergency room (19.3%) and 658 calls in the geriatric short stay (44.5%). Of the 1,480 calls for emergency hospital admission, 117 calls resulted in advice (7.9%), 346 calls resulted in deferred hospital admission (23.4%) and 15 calls resulted in simple consultations (1%). Among the 2,657 calls for advice/consultation/deferred hospital admission, 9.7% resulted in an emergency hospital admission.

**Table 1:** Synthesis of data collected from the questionnaire (caller, call recipient, patient age, reasons of calls, and degree of emergency)

	TOTAL (*n* = 4,137)	Call for advice (*n* = 2,657)	Call for hospital admission (*n* = 1,480)	p
	n (%)	n (%)	n (%)	
Caller				0.044
General practitioner	3,182 (76.9)	2,075 (78.1)	1,107 (74.8)	
Hospital physician	417 (10.1)	259 (9.7)	158 (10.7)	
Other (e.g. retirement home)	538 (13.0)	323 (12.2)	215 (14.5)	
Hotline responding physician				0.081
Assistant physisican	1170 (29.3)	727 (28.4)	443 (31.0)	
Hospital physician	2818 (70.7)	1833 (71.6)	985 (69.0)	
Reason for calling				-
Advice	1115 (27.0)	1115 (42.0)	0 (0)	
Emergency department hospitalisation	54 (1.3)	0 (0)	54 (3.6)	
Emergency geriatric department hospitalisation	1426 (34.5)	0 (0)	1426 (96.4)	
Consultation request	99 (2.4)	99 (3.7)	0 (0)	
Deferred hospitalization	1443 (34.9)	1443 (54.3)	0 (0)	
Age (median(interquartile))	87 (8)	87 (8)	87 (8)	0.470
Degree of emergency perceived by the hotline physician (mean(SD))	6 (3)	5 (3)	7 (2)	< 0.001
Degree of emergency perceived by the calling physician (mean(SD))	6 (3)	5 (3)	8 (2)	< 0.001

**Fig. 1:**
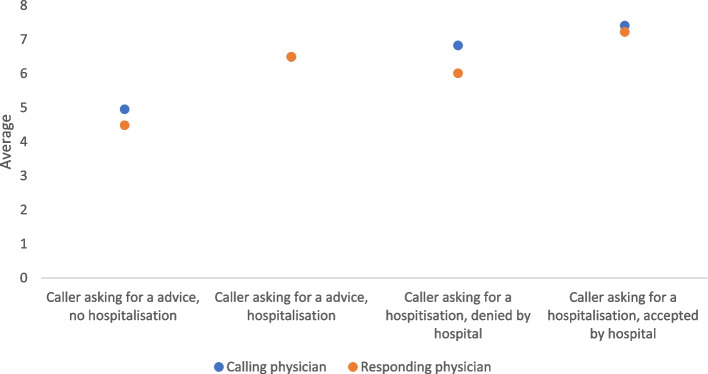
Degree of emergency by the caller and the respondent according to concordant/discordant groups

An analysis of the reason of call (request) and the solution offered for the request allow to classify the calls in 4 groups. Two concordant groups, call for advice and answer for advice and emergency call and answer for an emergency, represented respectively 58% and 22.8% of the hotline exchanges, 80.8% of the total. In opposition to concordant calls, we recorded 6.2% for call for advice and emergency response and 13% for an emergency call that resulted in an advice response, 19.2% in total of discordant calls. The degree of emergency showed significant differences between the four groups (see Fig. 1).

The averages of the degree of urgency for the concordant call groups were always higher for the caller than for the responding physician. For the discordant groups, the averages between the caller and the answering doctor varied. Means were identical for the discordant group of consultation and hospitalization responses. The difference in means between caller and responder for an emergency call and a non-emergency response was even higher than that for the concordant groups (*p* < 0.0001).

## Discussion

The study’s main objective was to investigate the potential effectiveness of hotlines in preventing inappropriate emergency room visits by older adults through the solutions provided by the responding physicians (geriatricians). Of the 1,480 calls for emergency hospitalization, 63.7% ended up in hospitalization in the emergency room or a geriatric short-stay. The hotline has therefore decreased by 36.3% the number of hospitalizations in the emergency room or a geriatric short-stay. Our results are consistent with those found in the literature on avoidance of emergency departments visits, ranging from 5 to 81% for different hotlines [23, 25–28]. The 36.3% decrease in emergency department admissions of older adults in our cohort echoes the 13–40% of inappropriate older adults emergency department admissions in other studies [16–19]. Of the 2,657 calls for advice/consultation/deferred hospitalization, 9.7% also resulted in emergency hospitalization.

Older adults spend more time in the emergency department than younger populations [29]. They often present complicated health issues, at times associated with functional and cognitive impairments [30, 31]. Emergency department visits are critical events for the older adults [32]. Moreover, emergency department admissions of older adults are independently associated with functional decline of their daily activities [13, 14]. These negative effects highlight the need of avoiding emergency room visits by the older adults. Hotlines calls showed the potential to reduce the use of emergency services through response offered by the geriatrician, that include deferred hospitalization, advice, or even a geriatric short-stay hospitalization instead of an emergency admission.

This study also highlighted the degree of concordance between the requests of the general practitioners and the answers of the responding physicians. Indeed, 80.8% of the calls were concordant between the initial request and the response provided. The response provided by the Hotline’s responding physicians confirmed the GPs’ initial diagnosis and confirmed their choice of the service offered. In addition, the Hotline responder offered consultation of a geriatrician to avoid unnecessary hospitalizations and alerted on an urgent situation not initially identified.

This study also allowed the analysis of a real aged population since the average age of the patients included was 86.8 years.

However, this study presents some limitations. In fact, the study sites enrolled in this multicentre research are of different sizes, structural organizations and technical platform. The responding hotline physician was the decision-maker to recommend or not an admission to the emergency department hospitalization or outpatient care. As well, the response of the hotline physician depended on the geriatric policies of the centre and the organization of its geriatric territory. These elements demonstrated that the answers provided were sites-dependent and could not be generalized for consensus management of older adults in the whole country. Moreover, despite the large number of subjects included, we did not have enough data to compare the cities between them. The health outcomes for the older adults were not collected following the offered solutions (e.g. readmission or later admission, quality of life, mortality). We only have access to data on inpatients. Also, the results of this study could have been strengthened by feedback from participating physicians. This study was done during the COVID-19 epidemic.

Measures to reduce hospitalization are not a priority in the organization of care in France. For example, mobile medico-social team use has increased since the COVID-19 crisis but remains limited [33]. Several cases of hospitalizations in emergency rooms are not decided from concertation and coordination of care pathway. It is for this reason that the Hotline, through its coordination and orientation role, can be a future tool for reducing hospital admissions. This study showed the importance of hotlines in the orientation of care and management of the older adults. The results showed the potential effectiveness of hotlines in avoiding unnecessary hospitalizations or in identifying cases requiring emergency hospitalization. However, hotlines are not perfect and depend on the health care system of the territory where they are located, the population, and the available infrastructures. Previous study conducted by our research team showed that the use of geriatric hotlines reduced the number of inappropriate emergency room visits [34], reflecting the good articulation between town medicine and the hospital.

Hotlines can help improve the care pathway for older adults and pave the way for future advances, such as new modalities of patient care, development of more appropriate responses for this population, and potentially reducing inappropriate emergency room visits.

### Supplementary Information

**Additional file 1.**

## Data Availability

The datasets used and/or analysed during the current study available from the corresponding author on reasonable request.
