# Supplementary material for: Decreasing hospitalizations through geriatric hotlines: a prospective French multicenter study of people aged 75 and above
Source: BMC Geriatr. 2023 Nov 28;23:783. doi: 10.1186/s12877-023-04495-9 (PMC10685561; doi:10.1186/s12877-023-04495-9)
Supplement: Supplementary file 1 — Additional file 1. [file 12877_2023_4495_MOESM1_ESM.docx]

## QUESTIONNAIRE FILLED OUT WHEN THE PHONE CALL IS RECEIVED BY THE GERIATRIC HOTLINE


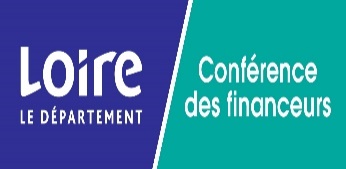

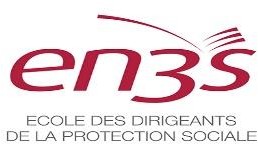


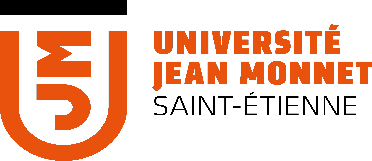

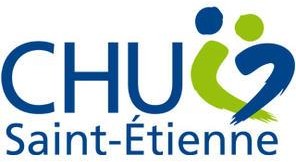


**Multicentre study on the impact of geriatric hotlines on the care pathways and health status of people aged 75 and over**

**Original data collection questionnaire to be filled out during the phone call by the hotline geriatrician**

**Place: …………………………**

**Date : _ _ / _ _ / _ _ _ _ Questionnaire N° : _ _**

**Caller**

- General practitioner □ Hospital physician □ Other ……………

**Hotline responding doctor**

□ Assistant physisican □ Hospital physician

**Reason for calling**

- Advice □ Consultation request
- Emergency department hospitalization □ Deferred hospitalization

**□** Emergency geriatric department hospitalization

**Patient age**: _ _ _ years old

## Degree of emergency perceived by the hotline physician

Scale from 0 to 10 (0: no emergency 🡪10: absolute emergency: …………/10

**Degree of emergency perceived by the calling physician**

Scale from 0 to 10 (0: no emergency 🡪10: absolute emergency: …………/10

## Response

- Simple advice

🡺 □ Médical □ Social-Medical □ Therapeutic

□ Emergency admission

🡺 □ Emergency department □ Short-stay geriatric care

If emergency admission: □ No beds in short-stay geriatrics □ Emergency care required

If Short-stay geriatric care: allocated bed: □ Yes □ No

- Deferred hospitalisation: ……. days
- Nonhospital ambulance services
- Outpatient care
- Consultation
- Teleconsultation
- Temporary nursing home

Call duration : …….. Minutes
